# Intermediate levels of vaccination coverage may minimize seasonal influenza outbreaks

**DOI:** 10.1371/journal.pone.0199674

**Published:** 2018-06-26

**Authors:** Veronika I. Zarnitsyna, Irina Bulusheva, Andreas Handel, Ira M. Longini, M. Elizabeth Halloran, Rustom Antia

**Affiliations:** 1 Department of Microbiology and Immunology, Emory University School of Medicine, Atlanta, GA, 30322, United States of America; 2 Department of Biological and Medical Physics, Moscow Institute of Physics and Technology, Dolgoprudny, 141701, Russia; 3 Department of Epidemiology and Biostatistics, University of Georgia, Athens, GA, 30602, United States of America; 4 Department of Biostatistics, University of Florida, Gainesville, FL, 32611, United States of America; 5 Vaccine and Infectious Disease Division, Fred Hutchinson Cancer Research Center, Seattle, WA, 98109, United States of America; 6 Department of Biostatistics, University of Washington, Seattle, WA, United States of America; 7 Department of Biology, Emory University, Atlanta, GA, 30322, United States of America; University of South Dakota, UNITED STATES

## Abstract

For most pathogens, vaccination reduces the spread of the infection and total number of cases; thus, public policy usually advocates maximizing vaccination coverage. We use simple mathematical models to explore how this may be different for pathogens, such as influenza, which exhibit strain variation. Our models predict that the total number of seasonal influenza infections is minimized at an intermediate (rather than maximal) level of vaccination, and, somewhat counter-intuitively, further increasing the level of the vaccination coverage may lead to higher number of influenza infections and be detrimental to the public interest. This arises due to the combined effects of: competition between multiple co-circulating strains; limited breadth of protection afforded by the vaccine; and short-term strain-transcending immunity following natural infection. The study highlights the need for better quantification of the components of vaccine efficacy and longevity of strain-transcending cross-immunity in order to generate nuanced recommendations for influenza vaccine coverage levels.

## Introduction

Influenza A remains a widespread disease that continues to circulate worldwide with more than 200,000 people in the US hospitalized annually for illnesses associated with seasonal influenza virus infections [1]. Vaccination is considered to be a chief preventive tool against influenza, and the Centers for Disease Control and Prevention encourages maximal vaccination coverage by recommending that all individuals 6 months and older receive an influenza vaccine annually [2].

The influenza vaccine in general use is an inactivated virus vaccine that targets the strains of influenza A and B predicted to circulate in the population during the current season. Many studies have measured the efficacy of the vaccine (we use “efficacy” to include estimates from both randomized and observational studies). Early studies of influenza vaccine efficacy in the military [3], as well as Tecumseh and Hutterite communities studies [4, 5] and studies in Japan [6] showed that the inactivated influenza vaccine provides reasonable levels of protection against the current circulating strain of influenza and may interrupt virus transmission. Meta-analyses in ten randomized controlled trials revealed pooled efficacy of trivalent inactivated vaccine (TIV) as 59% (95% CI 51-67) [7], while for one of the component of TIV vaccine, influenza A subtype H3N2, a meta-analysis of 56 test-negative design studies reported a much lower pooled vaccine efficacy of 33% (95% CI 26-39) [8]. The main challenge in vaccination against influenza is that antigenic drift of the virus in response to population level immunity generates virus strains that are not covered by the vaccine, and this requires frequent reformulation of the vaccine to include the drifted strains.

**Fig 1:**
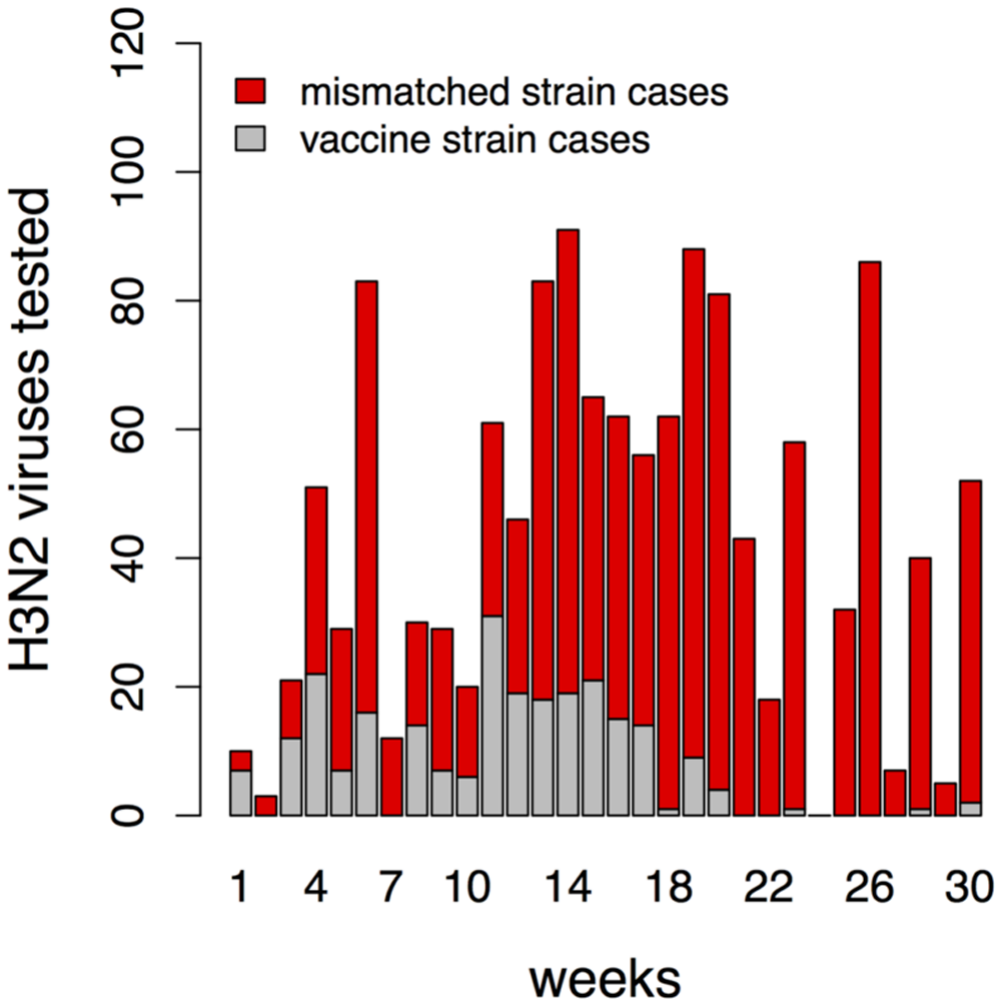
Influenza A (H3N2) strain replacement during season 2014-2015 in the US. The number of influenza A (H3N2) viruses tested that are similar to the H3N2 vaccine strain (A/Texas/50/2012) are shown in grey, and the number of cases caused by viruses with reduced titers to antiserum raised against the vaccine strain is shown in red. The weekly counts began October 26, 2014 [10].

Vaccination against H3N2 subtype typically shows lower vaccine efficacy in comparison to H1N1, so we will focus on H3N2. If during a given season only one strain, drifted from a previous season strain of a given subtype, would be in circulation, the situation would be relatively simple. In reality, phylogenetic analysis of HA genes from circulating H3N2 strains revealed extensive genetic diversity with multiple clades and subclades co-circulating (see CDC report for the current 2017-2018 season [9]). Multiple co-circulating strains generate potentially complex dynamics with ongoing replacement of older strains by newer ones. Consider, for example, the spread of influenza A (H3N2) in the US during the 2014-2015 season, where the H3N2 subtype dominated. Cases analyzed just prior to the onset of that influenza season showed that 42% were caused by strains antigenically similar to the strain included in that season’s influenza vaccine [10]. Characterization of the limited number of confirmed H3N2 cases during the seasonal outbreak is shown in Fig 1. We see that the fraction of H3N2 cases caused by the vaccine-like strains gradually declined, and the proportion of cases due to strains not targeted by the vaccine increased. By the end of the season, virtually all new cases were due to strains not targeted by the vaccine, and over the entire season 81% of the cases were caused by non-vaccine strains [11].

The circumstance described above is not unique. For example, by the final week of the 2007-2008 influenza season in US, 21% of reported cases were caused by the H3N2 strain included in the vaccine, 65% cases were caused by its known antigenic variant, and 14% showed somewhat reduced titers to antisera produced by any of these two strains [12].

To better understand the nature of the phenomenon described above, we briefly discuss how influenza varies and the epidemiological and immunological consequences of this variation. Infection with a given strain of influenza results in the generation of a long-lived antibody response specific for hemagglutinin (HA) and neuraminidase (NA), the virus surface proteins that are the major targets of the humoral immunity [13]. The influenza virus responds to preexisting immunity in the host population by antigenic changes through mutations in HA and NA. In addition to long-lived strain-specific immunity mentioned above, infection induces strain-transcending immunity that is of a shorter duration. This short-term strain-transcending immunity has been demonstrated in a number of immunological studies and is also invoked in epidemiological studies that describe the circulation of different influenza strains. Immunological studies in mouse and ferret model systems have shown that infection with one strain induces temporary strain-transcending immunity with a relatively short duration on the scale of one or a few months [14–19]. The mechanisms for this strain-transcending immunity, or cross-immunity, following natural infection are the subject of much current work and could arise due to temporary non-specific antiviral immunity [14] or T cells specific for internal epitopes which are typically conserved between strains [15–19]. In contrast to natural infection, the trivalent inactivated vaccine (TIV) primarily induces humoral strain-specific immunity directed against HA and NA included in the vaccine and does not generate broad cross-immunity against mismatched strains. Epidemiological studies also indicate the presence of temporary strain-transcending immunity [20–23]. Observations of infection of humans over the course of a single season show that infection with one strain significantly reduces the risk of infection with an antigenically different strain [23]. Short-term cross-immunity was used to explain the limited diversity of circulating influenza strains [20, 21], though other studies suggest alternative explanations [24–26].

We extend prior studies that use mathematical models to analyze the dynamics of pathogens exhibiting strain variation [22, 27–31]. We extend these models to analyze how the size of seasonal outbreaks of influenza depends on vaccination coverage in a simple scenario with two antigenically distinct co-circulating strains and vaccination which targets one of these strains. This allows us to explore how the optimal vaccination strategy depends on factors such as the initial relative prevalence of the two strains and the duration of cross-immunity generated by natural infection with influenza. Our results suggest that high levels of influenza vaccination coverage may lead to the replacement of the dominant strain targeted by the vaccine with another co-circulating mismatched strain that would otherwise be suppressed from the cross-immunity generated in the population by natural infection with the dominant strain. We show that increasing vaccination coverage above an optimal level may, in fact, lead to a higher total number of influenza cases (attack rate).

The goal of this study is not to come up with definitive recommendations for vaccination policy, but to explore the complex consequences of vaccination for pathogens that exhibit strain variation, with a focus on influenza. In doing so, we bring attention to possible unexpected consequences of high vaccination coverage, and identify key factors that need to be evaluated when considering the optimal vaccine coverage that minimizes the attack rate from influenza.

## Model

The model incorporates the following biological features: (i) two co-circulating antigenically distinct influenza strains; (ii) natural infection with either strain induces long-term immunity specific for that strain and short-term strain-transcending immunity (i.e. cross-immunity) to the other strain; (iii) vaccination induces protection only against the strain included in the vaccine.

**Fig 2:**
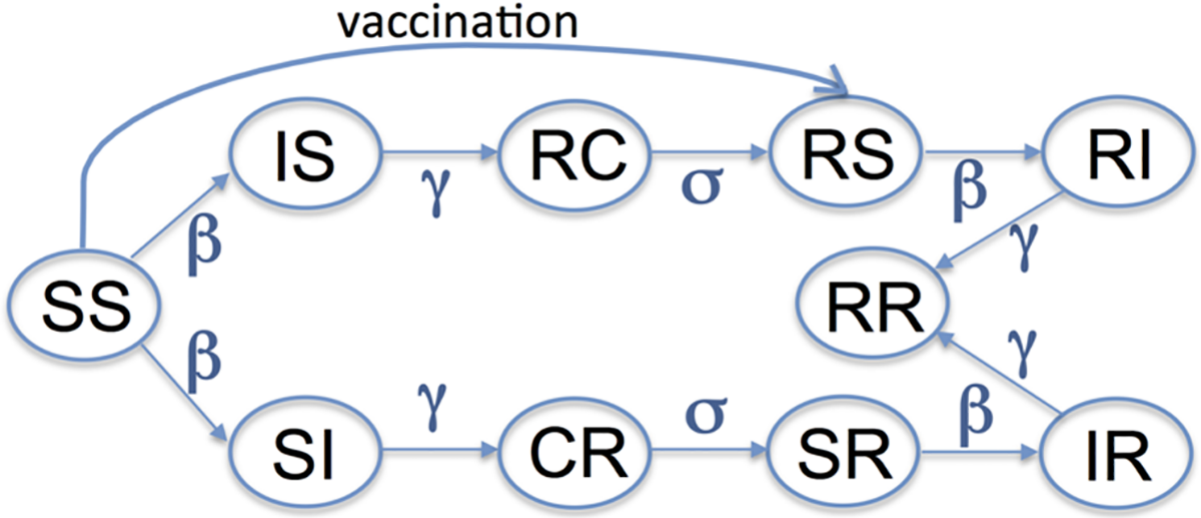
Scheme for SIR-based model with two strains. Ten different states corresponding to model variables in eqs (1)–(10) characterize the status of individuals with respect to the first and second strains of the virus. The first and second letters in the two-letter code show the individual’s status of infection with respect to the first and second strain. We use the conventional notation “S” for susceptible, “I” for infected, and “R” for recovered with long-term immunity and “C” for recovered with short-term cross-immunity.

Our model is based on the widely used Susceptible-Infectious-Recovered (SIR) framework for disease transmission, which puts individuals in the population into compartments based on their immune and infection status [32–34]. The SIR framework has been extended to model strain variation in influenza in a number of different ways [20, 22, 26, 35–37]. We incorporate two co-circulating influenza strains using a status-based approach (Fig 2) [22, 35]. A two-letter code is used to describe the ten possible states for each individual in the population, where the first and second letters reflect the status with respect to the first and second influenza virus strains. For example, *SS* denotes individuals susceptible to the infection with both strains and *SR* represents an individual susceptible to the first strain but recovered from the second strain and immune to it. Infection of susceptible individuals (*SS*) with the first strain moves them to the *IS* state at a rate that depends on the infectiousness of the first strain and the prevalence of infection caused by it. Infectious individuals recover and move to *RC* state, which indicates that they have long-term immunity to the first strain (*R*) and short-term cross-immunity (*C*) to the second strain. These individuals lose immunity to the second strain at rate *σ* and move to the *RS* state. Individuals in the *RS* state can be infected only by the second strain, and this would result in movement to the *RI* state and, subsequently, after recovery, to the *RR* state. We note that the state space variables are the fractions of the total population in each state and, consequently, we are not modeling a population of any particular size. The model variables and parameters are summarized in Table 1. We chose the basic reproductive number of seasonal influenza to be 1.6 [38, 39]. The average duration of infection (reciprocal of *γ*) was defined as the viral shedding period of an infected adult and was set to 5 days [40]. The duration of cross-immunity was varied from 60 days to 1 year [20].

**Table 1:** Model parameters and initial values for variables.

**Model parameter**	**Symbol**	**Value**
Basic reproduction number	*R*_0_	1.6 [38, 39]
Transmission coefficient	*β* day^−1^	*R*_0_ × *γ*
Recovery rate	*γ* day^−1^	1/5 [40]
Rate of loss of cross-immunity	*σ* day^−1^	1/365 − 1/60 [20]
Vaccine efficacy	*V*_*E*_	0.5
Fraction vaccinated	*f*	0 − 1
**Model variable**	**Symbol**	**Initial condition**
Susc. to both strains	*SS*	1 − *IS* − *V*_*E*_ *f*
Inf. with strain 1, susc. to strain 2	*IS*	2 10^−4^
Susc. to strain 1, inf. with strain 2	*SI*	2 10^−4^ @ *t* = Δ*T*
Time of introduction of strain 2	Δ*T* day	0 < Δ*T* < 100
Rec. from str. 1, cross-imm. to str. 2	*RC*	0
Rec. from str. 2, cross-imm. to str. 1	*CR*	0
Rec. from str. 1, susc. to str. 2	*RS*	0
Susc. to str. 1, rec. from str. 2	*SR*	0
Inf. with str. 1, rec. from str. 2	*IR*	0
Rec. from str. 1, inf. with str. 2	*RI*	0
Rec. from both strains	*RR*	0

The parameters for the simulations are indicated above unless otherwise specified in the figure legend.

The model equations are:

$$\begin{array}{ccc} dSS/dt & = & -\beta (IS+IR+SI+RI)SS \end{array}$$ (1)

$$\begin{array}{ccc} dIS/dt & = & \beta (IS+IR)SS-\gamma IS \end{array}$$ (2)

$$\begin{array}{ccc} dSI/dt & = & \beta (SI+RI)SS-\gamma SI \end{array}$$ (3)

$$\begin{array}{ccc} dRC/dt & = & \gamma IS-\sigma RC \end{array}$$ (4)

$$\begin{array}{ccc} dCR/dt & = & \gamma SI-\sigma CR \end{array}$$ (5)

$$\begin{array}{ccc} dRS/dt & = & \sigma RC-\beta (SI+RI)RS \end{array}$$ (6)

$$\begin{array}{ccc} dSR/dt & = & \sigma CR-\beta (IS+IR)SR \end{array}$$ (7)

$$\begin{array}{ccc} dRI/dt & = & \beta (SI+RI)RS-\gamma RI \end{array}$$ (8)

$$\begin{array}{ccc} dIR/dt & = & \beta (IS+IR)SR-\gamma IR \end{array}$$ (9)

$$\begin{array}{ccc} dRR/dt & = & \gamma (IR+RI) \end{array}$$ (10)

The two strains have similar parameters except for their initial prevalences that determine the degree of initial dominance of the first strain. We changed the degree of dominance by altering the time of introduction (Δ*T*) of the second strain. Higher values of Δ*T* result in a higher degree of dominance of the first strain. An alternative way of changing the degree of dominance is to introduce the two strains simultaneously (at *t* = 0) at different frequencies. We have shown that it did not alter the qualitative outcomes of the model (see Model Robustness section).

Because the duration of one epidemic season is short, we did not include births and deaths or migration in our model. Different studies showed a wide range in estimated vaccine efficacy (16 to 76% for adults 18-64 years old, see Table 2 in [7]). In our model, we assume that the vaccine provides all-or-none protection from infection, and define the vaccine efficacy (*V*_*E*_) as the fraction of individuals who are protected. We introduced vaccination by moving *fV*_*E*_ of individuals from *SS* to *RS* prior to the season, where *f* is a fraction of vaccinated people. We assume that infection, but not vaccination, induces short-term strain-transcending immunity—this is consistent with the shift from the vaccine strain to the mismatched strain over the course of the season shown in Fig 1.

We first considered the case where individuals are resistant to superinfection, i.e. individuals can only be infected by a single strain at a given time. We then explored the robustness of our model by considering scenarios that include superinfection, simple age-structure and where infection results in long-term cross-immunity in a fraction of individuals (see Figs A-C in S1 Text).

## Results

### Dynamics of strain competition

**Fig 3:**
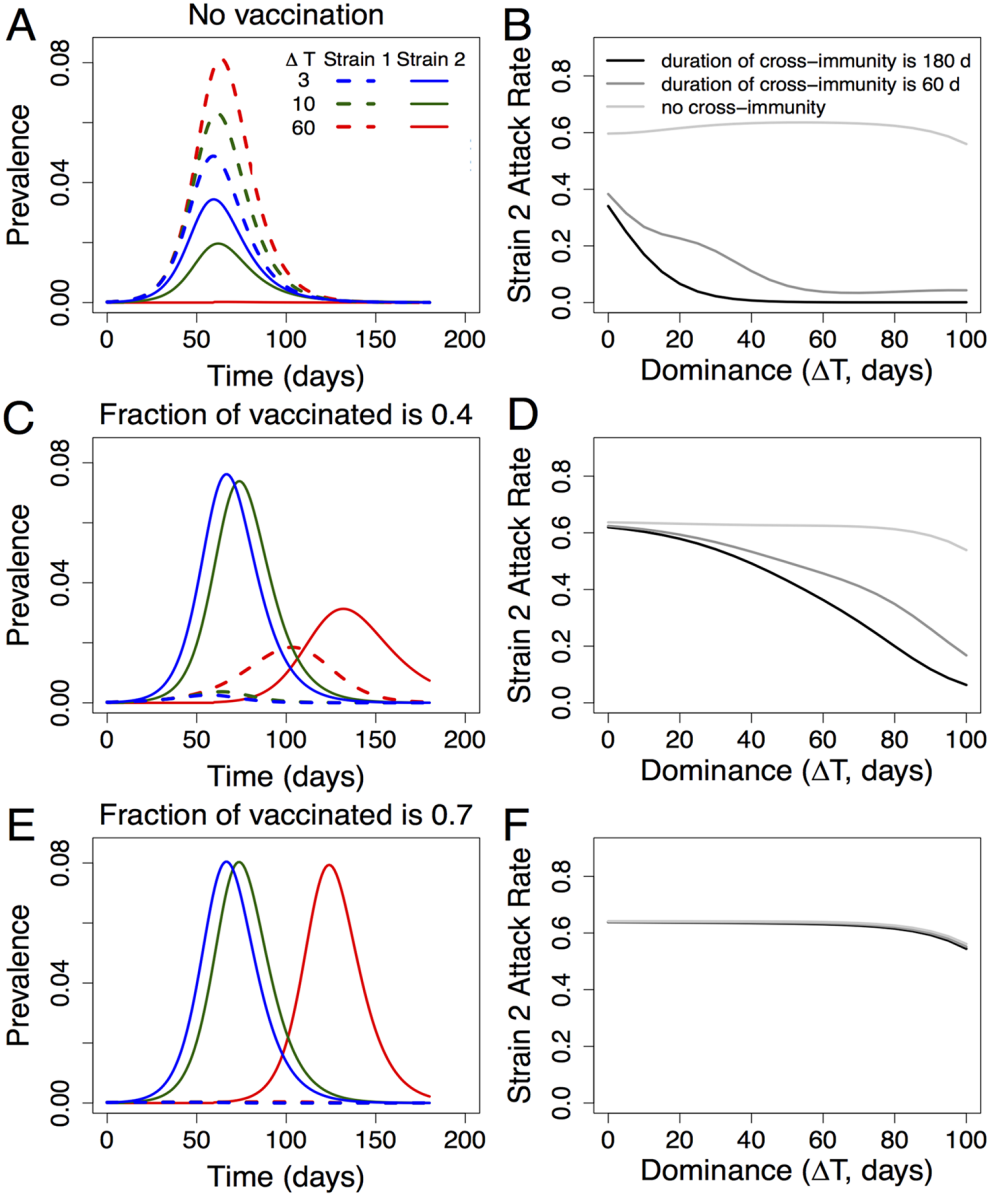
The dynamics of circulation and competition between the two strains of influenza depends on the initial dominance of the first strain (Δ*T*, the time between introduction of the first and second strains), the duration of cross-immunity (1/*σ*) and the extent of vaccination. Panels A and B show competition between the two strains in the absence of vaccination. Panel A shows that the extent to which the first strain (dashed lines) suppresses the second strain (solid lines) increases with an increase in initial dominance (Δ*T* = 3, 10, 60 shown in blue, green and red). Panel B shows that the suppression of the second strain increases with the degree of initial dominance of the first strain (Δ*T*) and duration of cross-immunity (1/*σ*). Panels C and D show that increasing the level of vaccination against the first strain reduces its prevalence and the extent to which it suppresses the second strain. The solid lines in Panel C are higher than in Panel A, and the attack rate of the second strain in Panel D is higher than in Panel B. Panels E and F show that when the level of vaccination is high the strain replacement occurs—the first strain is eliminated by vaccination and replaced by the second strain. In the left Panels (A, C, E) the duration of cross-immunity 1/*σ* = 180 days, and other model parameters are in Table 1.

We consider the dynamics of two co-circulating influenza strains and how it is changed by vaccination. Fig 3 shows the dynamics of two strains of influenza and how it is affected by the degree of initial dominance of the first strain, the duration of cross-immunity and the extent of vaccination. The degree of dominance was modeled by delaying the time of introduction of the second strain by Δ*T*. The prevalence of a given strain was defined as a fraction of the total population that is infected with that strain and is shown by the dashed and solid lines for the first and second strains, respectively.

Competition between the two strains is mediated by cross-immunity induced following infection with either strain. Fig 3 Panel A illustrates that in the absence of vaccination, the extent to which the first (dominant) strain can suppress the second strain increases with the extent of initial dominance of the first strain (i.e. suppression of the second strain is higher when it is introduced later). Fig 3 Panel B shows in more detail how the suppression of the second strain depends not only on the degree of initial dominance of the first strain but also on the duration of cross-immunity (1/*σ*). The attack rate of the second strain (i.e. the total number of cases with the second strain) during the outbreak is reduced by increases in the degree of dominance of the first strain Δ*T* from 0 to 100 days, and if we increase the duration of cross-immunity 1/*σ* from 0 to 180 days. For Fig 3 Panels B, D, and F, the attack rate is calculated for one influenza season which we assume to last for about six months (180 days in our simulations). In the absence of competition between the two strains (light grey lines for “no cross-immunity” condition), the attack rate for the second strain does not depend on the vaccination against the first strain (Fig 3 Panels B, D, and F).

As the level of vaccination against the dominant strain increases, its prevalence decreases and, consequently, the extent to which it suppresses the second strain falls. This is seen in Fig 3 Panel C, where the size of the outbreaks due to the second strain is much higher than the corresponding outbreaks in the absence of vaccination shown in Fig 3 Panel A. Finally, when the vaccination coverage is sufficiently high, it eliminates the outbreak with the first strain, and it is replaced by the outbreak with the second strain (Fig 3 Panel E). In this case, the magnitude of the outbreak with the second strain becomes independent of both the degree of dominance of the first strain and the level of infection-induced cross-immunity (Fig 3 Panel F). The timing of the outbreak depends on the time of introduction of the second strain (Fig 3 Panel E).

### Vaccination changes the total attack rate

**Fig 4:**
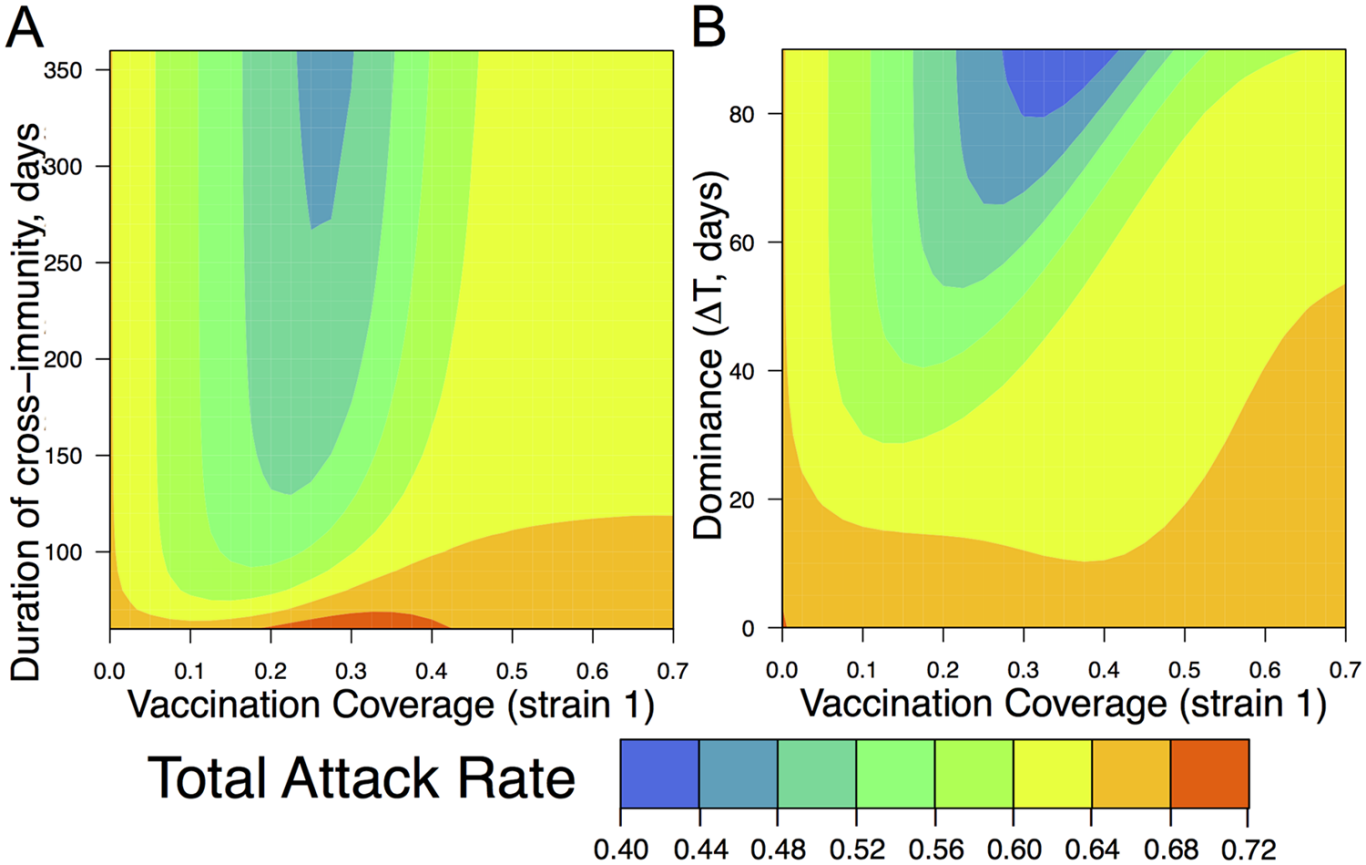
The total attack rate depends on the vaccination coverage, the duration of infection-induced cross-immunity (1/*σ*) and the degree of dominance (Δ*T*). Panel A shows how the total attack rate depends on the level of vaccination coverage and the duration of cross-immunity (*σ*). The time of introduction to the second strain is kept fixed at 60 days. Panel B shows how the total attack rate depends on the vaccination coverage as well as the degree of dominance (the time delay Δ*T* between introduction of the first and second strains). The parameter for the duration of cross-immunity after influenza infection 1/*σ* = 180 days. All other model parameters are in Table 1.

Fig 3 shows that the dynamics of strain competition is affected by three key parameters: (i) extent of vaccination coverage; (ii) duration of cross-immunity (1/*σ*); and (iii) degree of initial dominance of the first strain (Δ*T*). We would like to know how the total attack rate due to influenza is affected by the level of vaccination coverage. The total attack rate is the sum of the attack rates with the first and the second strains over the season. In Fig 4A the degree of dominance of the first strain is kept constant (the second strain is introduced at day 60) and the total attack rate is plotted as a function of the vaccination coverage against the first strain and the duration of cross-immunity 1/*σ*. In Fig 4B the duration of cross-immunity is held constant (at 1/*σ* = 180 days) and the total attack rate is plotted as a function of the extent of vaccination and the degree of dominance (i.e. the time delay Δ*T*).

Increasing the vaccination coverage initially decreases the total attack rate. However, for a large parameter regime, after a critical level of vaccination, further increases in the vaccination coverage lead to an increase in the total attack rate. The initial decrease in the attack rate is expected because of the decrease in the prevalence of the dominant strain that is targeted by the vaccine. The subsequent increase in the total attack rate at high levels of vaccination coverage occurs because of strain replacement described in Fig 3. The feedback between the prevalence of each strain and the extent of competitive suppression between the two strains determines the magnitude of the total attack rate and the level of vaccination coverage at which the total attack rate is minimized (see Discussion on the “overshoot” effect).

This result (i.e. the total attack rate being minimized at intermediate levels of vaccination against one strain) does not require cross-immunity induced by infection to be complete. Previous studies considered a range for the duration of cross-immunity between three months and two years [20], and our results hold for an even wider range of cross-immunity as shown in Fig 4A. This result also requires a degree of dominance Δ*T* > about 20 days (Fig 4B), and vaccination against the dominant strain. If we assume that at the early stages the dominant strain grows exponentially, then a time delay Δ*T* ≈ 20 corresponds to the case where the first strain would have a relative prevalence of *e*^(*R*_0_*S*−1)Δ*Tγ*^ ≈ 10 fold higher than that of the second strain.

### Optimal vaccination coverage

**Fig 5:**
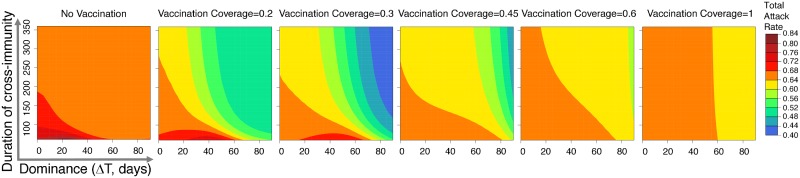
Effect of different levels of vaccination coverage on the total attack rate. Each panel shows how the total attack rate depends on the duration of cross-immunity and the degree of dominance (Δ*T*) for a given level of vaccination. The level of vaccination increases from left to right. All other model parameters are in Table 1.

Finally, we asked how the optimal level of vaccination coverage depends on both the level of cross-immunity as well as the time of the second strain’s introduction. Each panel in Fig 5 corresponds to a different level of vaccination and shows how the total attack rate depends on the level of cross-immunity and the degree of dominance. For a given level of cross-immunity and dominance (i.e. position on the graph) we can see how changing the level of vaccination changes the attack rate by comparing the attack rate moving from the plots on the left (low vaccination) to the plots on the right (high vaccination).

**Fig 6:**
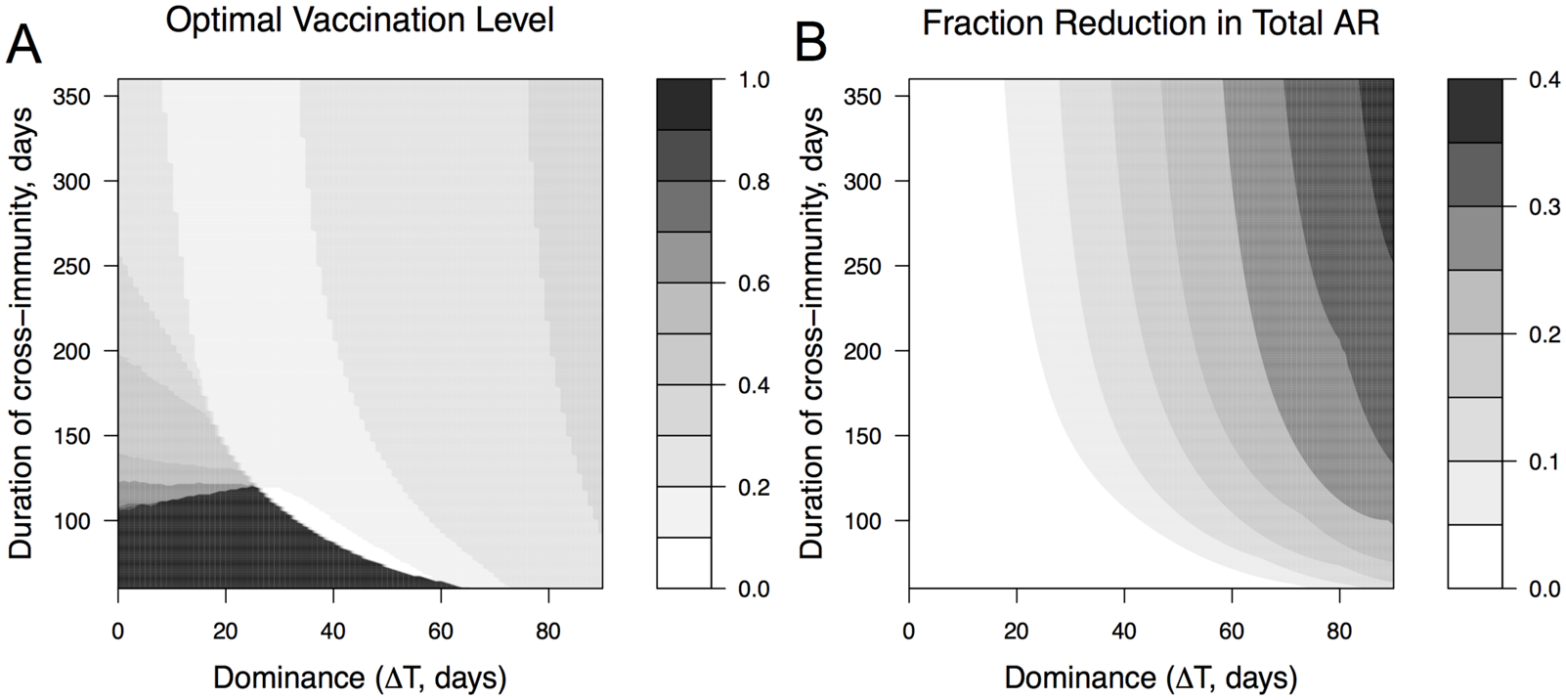
Optimal vaccination level and the extent to which it reduces the total attack rate. Panel A shows the optimal vaccination rate that minimizes the total attack rate and how it depends on the degree of dominance (time of introduction of the second strain) and duration of cross-immunity. Panel B shows the corresponding reduction in total attack rate calculated as the difference between the total attack rates at maximal coverage and optimal coverage divided by the total attack rate at the maximal coverage. All other model parameters are in Table 1.

Fig 6A plots the optimal vaccination level, and Fig 6B shows how much benefit it produces in comparison to the case of complete vaccine coverage. From Fig 6A we can see that complete vaccination coverage is desirable only if the duration of cross-immunity is very low and provided that the dominance Δ*T* of the first strain is less than 60 days. For a substantial region of parameter space we find that a relatively modest vaccination coverage (between 10 and 50%) is optimal. From Fig 6B we also can see that for a large parameter regime there is only a modest difference in the outcome with the degree of vaccination coverage. The benefit of optimal vaccination vs vaccinating the total population can be relatively large (between 25-40%) when the cross-immunity and dominance is relatively high.

### Model robustness

We have checked the robustness of our model by determining how the results are affected by introduction of a number of changes. First, we added superinfection to the model and consider the opposite limiting case by allowing someone infected with one strain (*IS* or *SI*) to be infected with the other strain (become *II*) at the same rate *β* as the *SS* individuals (Fig A in S1 Text). Contrasting the two extreme scenarios for incorporating superinfection into the model, we demonstrated that superinfection does not play a significant role in the observed dynamics (Fig D in S1 Text). Second, we added heterogeneity to the model by modifying it to include two age groups, children and adults, with higher levels of transmission and slower recovery for children (Figs B and E in S1 Text). This allowed us to incorporate a higher level of transmission in children and lower level of transmission in adults as has been described earlier [41]. Third, we changed the structure of our model to incorporate a different formulation for cross-immunity. In the current model, after recovery from natural infection, the level of cross-immunity gradually wanes. We changed this to allowing a fraction of hosts to have permanent cross-immunity after infection with either strain (Figs C and F in S1 Text). Fourth, we added seasonality into the model as has been previously done [22] by changing the parameter *β* as a function of time so that *β*(*t*) = *β*(1 + *β*_*s*_sin(2*πt*/365)) (Fig G in S1 Text). Fifth, we changed the way we introduced the two strains. Previously we introduced the subdominant strain at a later time, and here we considered the effect of introducing the two strains at the same time (at *t* = 0) but at different frequencies (Fig H in S1 Text). Sixth, we changed the *R*_0_ to a higher value which might occur following larger antigenic changes in the virus. In Fig I S1 Text we show that increasing *R*_0_ from 1.6 to 2.0 did not alter the main qualitative result—the optimal reduction in the attack rate occurs at intermediate levels of vaccination, though there was a modest increase in the optimal level of vaccination coverage. Seventh and finally, we considered a scenario where there were additional strains not targeted by the vaccine. It can be shown that if cross-immunity lasts for the duration of a season then the multi-strain model can be reduced to our two strain model with the multiple strains which are not targeted by the vaccine combined together. The more complex scenario where cross-immunity does not last an entire season is illustrated for a three strain model in Figs J and K S1 Text. The qualitative results are similar to those of the two strain model shown in Fig 4.

The details of these changes and corresponding models equations are shown in S1 Text. We note that none of the changes or additions to the model altered the qualitative results, indicating the robustness of the basic results of our model.

## Discussion

We considered the dynamics of two co-circulating influenza strains, and how it is changed by vaccination. This allowed us to explore how vaccination affects the total attack rate, and we showed that an intermediate level of vaccination minimizes the total attack rate. We also showed that our simple model is robust to inclusion of complexities such as superinfection, seasonality, and heterogeneity in transmission in subpopulations, as well as a different way of describing strain dominance.

We explored the problem of vaccination against pathogens, such as influenza, that exhibit ongoing strain variation. Our study builds on previous work on vaccination and strain replacement in *Streptococcus pneumoniae* [28], and multi-strain models of influenza [22]. The model of Lipsitch [28] describes the situation when vaccination against the targeted serotypes may cause an increase in carriage of non targeted serotypes in the case of *S. pneumoniae* and *Haemophilus influenzae*, which have multiple well defined strains and relatively slowly changing strain structure compared with influenza. The potential for large-scale vaccination campaigns to alter the competitive landscape between influenza subtypes has been mentioned before [42], and we integrate and expand on all of these earlier studies.

The model makes the novel prediction that increasing influenza vaccination coverage beyond an optimal level may lead to a higher total attack rate. The finding that the maximal level of vaccination coverage may not always be the optimal solution has been reported previously in other very different scenarios [43, 44].

**Fig 7:**
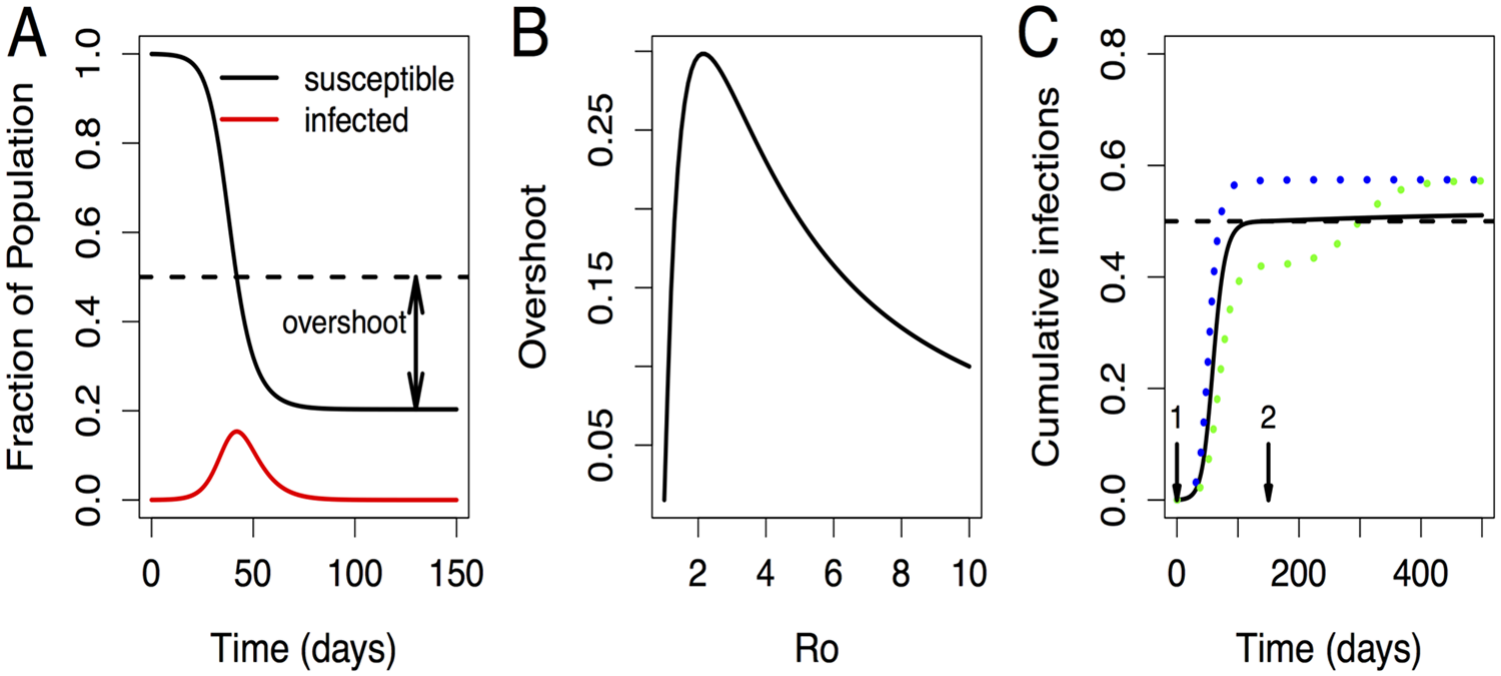
Illustration of the “overshoot” effect. Panel A shows the number of susceptibles and infecteds during an outbreak with *R*_0_ = 2. The initial growth phase of the epidemic is approximately characterized by an exponential increase in the number of infecteds, accompanied by a decline of susceptibles. The dashed horizontal line indicates the threshold level of susceptibles below which population immunity prevents further outbreaks for this given set of parameters. Once the number of susceptibles crosses a threshold level, the average number of new infections caused by an infected person falls below 1 and the epidemic wanes. The arrow indicates the difference between the number of susceptibles at the end of the outbreak and the threshold line. This difference was termed as the “overshoot” [45]. Panel B shows how the magnitude of overshoot depends on *R*_0_. For the simple SIR model, the number of prevented infections is found to be highest for intermediate values of *R*_0_ ∼ 2. Panel C considers the optimal level of vaccination for the limiting case which is easy to analyze. We choose an infection with *R*_0_ = 2, high degree of dominance (second strain is introduced at day 150) and high cross-immunity which does not wane on this timescale. We plot the cumulative number of infections by either strain (as a fraction of the total population) as a function of time. The optimal level of vaccination (fraction vaccinated = 0.209, solid black line) against the first strain results in the fraction of susceptibles to the second strain declining to ∼1 − 1/*R*_0_ = 0.5. This level of herd immunity just prevents the second strain from spreading. At a lower level of vaccination (fraction vaccinated = 0.16, blue dotted line) leads to more infections with the first strain (and also prevents the second strain spreading). A higher level of vaccination (fraction vaccinated = 0.26, green dotted line) leads to fewer infections with the first strain but allows the second strain to spread and generate an overshoot.

Our result for the optimal level of vaccination can be intuitively explained with the help of the *“overshoot”* effect [45] that describes the observation that the total attack rate of an epidemic is much larger than the fraction of immune or recovered individuals needed to prevent an epidemic (Fig 7 Panels A and B). Consider the limiting case when the degree of dominance is high and short-term cross-immunity is high. In this case, the optimal level of vaccination for the first strain is such that the number of individuals recovered from infections with the first strain (1 − 1/*R*_0_) is just sufficient to prevent the second strain from spreading (and this eliminates the “overshoot”) (Fig 7 Panel C). The situation is more complex when the degree of dominance is lower but the basic result is that a single large outbreak (either with a vaccine-matched strain in the absence of vaccination or the mismatched strain when the vaccine prevents the outbreak of the vaccine-matched strain) can result in a greater attack rate than the total attack rate from a smaller outbreak of the vaccine-matched strain that generates sufficient cross-immunity to blunt the subsequent outbreak with the vaccine mismatched strain. Similar scenarios can also occur for the use of antivirals and drug sensitive and resistant strains [46–48]. At the optimal level of vaccination, the effective reproductive numbers and overshoot are reduced for both strains. This occurs because of direct targeting of the dominant strain by vaccination and indirect targeting of the second strain by strain-transcending immunity after influenza infection.

We now discuss the assumptions and limitations of our model, and how the model would need to be extended to accurately capture the dynamics of circulating influenza strains. The first set of assumptions is that influenza exhibits strain variation, and that multiple strains co-circulate at the same time. As discussed in the introduction, this is not uncommon for the H3N2 influenza subtype. The model is a relatively simple deterministic compartmental model and includes only two influenza virus strains of a given subtype, one which is targeted by the vaccine and one which has substantially reduced titers to the immune response induced by vaccination. In reality, there is likely to be a number of strains with a more graded response to vaccine-induced immunity. If there are only two strains, then it might be best to include both strains in the vaccine, and this has been suggested by [49]. However, we would expect the problem described here to re-emerge, if there is an additional strain not targeted by the vaccine, and this is regardless of the number of strains included in the vaccine. In such a scenario, we would once again expect the lowest total attack rate to happen at an intermediate level of vaccine coverage, and complete vaccine coverage to be counterproductive. If only one strain is included in the vaccine, addition of more not targeted by a vaccine strains will not change the result qualitatively, as described in the section on the robustness of the model and illustrated in Figs J and K in S1 Text.

Another set of assumptions concerns the specificity and longevity of immunological memory induced by vaccination vs. natural infection. The model assumes that both natural infection and vaccination generate immunity that lasts at least the entire season to the specific strain included in the vaccine and, thus, in the model this immunity does not wane in this period. We additionally assume that natural infection, but not vaccination with the inactivated virus such as TIV, generate shorter-term strain-transcending immunity. These assumptions are similar to those used in earlier models [20, 22]. Our model covers a broad range for the longevity of cross-immunity lasting between two months to over a year as in a previous study [20]. Both innate immunity [14] and CD8 T cell responses to conserved virus epitopes [15–19] could provide strain-transcending immunity following influenza infections. Innate immunity, however, would be expected to last for a relatively short-duration of a few weeks [50], while elevated levels of resident memory T cells may be maintained in the respiratory tract for months [19], and would be more relevant to short-term cross-immunity [51–53].

We assume that vaccine generates all-or-none immunity that renders half vaccinated individuals resistant to infection. Changing *V*_*E*_ does not change the qualitative results but just scales how the results depend on vaccine coverage *f*, and this is because the results depend on the product *fV*_*E*_. If we were to use a “leaky” vaccine model, then we would also need estimates of the effect of the vaccine on infectiousness and pathogenicity, which are other components of the efficacy of the vaccine, and are not readily available. An important next step will be to obtain more accurate estimates of these different components of vaccine efficacy and incorporate them into the model.

Going from the simple qualitative results of our paper to quantitatively accurate predictions will require more detailed models such as individual based models which incorporate more complex and realistic contact networks, spatial structure, and stochasticity. These models will need to account for seeding of seasonal outbreaks and a quantitative understanding of cross-immunity between the responses to different viruses [54–57]. The models assume all individuals in the population have identical immunity generated by exposure to influenza in the past. They will also need to taking into account the prior immune history to influenza infection that may lead to mild and/or asymptomatic infections, mild infection as a result of the vaccination and effect of influenza vaccine on secondary bacterial infections [58–61]. Finally, we have assumed that there is no inherent fitness difference in the two strains (in the absence of immunity) and need to consider the process of generation of variation and how vaccination can change the tempo of antigenic evolution which has been considered in a recent paper [62].

The paper uses simple conceptual models to explore the consequences of the increased vaccination coverage on the dynamics of the co-circulating strains and total influenza attack rate. The surprising prediction of the model is that for a wide range of biologically reasonable parameters the maximum reduction in the total attack rate occurs at the intermediate level of vaccine coverage, and, somewhat counter-intuitively, further increasing the level of the vaccination coverage may lead to higher number of influenza infections and be detrimental to the public interest. Bringing the models predictions into contact with epidemiological data will be the subject of subsequent studies. It will require taking into account factors such as the match between the vaccine and the dominant strain and the frequencies of vaccine matched and mismatched strains at the beginning of the season, as well as more realistic contact networks and other factors mentioned in the previous paragraph.

While we focus on antigenic drift, the model can also be applied to a scenario involving an antigenic shift that causes pandemics. This is because of the high degree of conservation of T cell epitopes on influenza virus internal proteins not only for strains of a given subtype but also for strains of different virus subtypes [63, 64]. In this case, vaccination which reduces the prevalence of the seasonal strain may decrease the level of cross-immunity generated by infection with a seasonal strain and, thus, weaken a protection against the shifted (pandemic) strain. As pandemic strain often has higher transmissibility in comparison to a seasonal strain [65], vaccination may facilitate its spread. This might suggest that vaccination against the currently circulating seasonal influenza should be halted following the emergence of a pandemic strain, which is consistent with the report that people vaccinated against seasonal influenza strain had a higher rate of infection with pandemic strain and more severe disease during that season [66]. It is also consistent with the study showing that infection with H3N2 was negatively associated with a subsequent risk of infection with H1N1 during the same influenza season [23].

In summary, our models suggest that more detailed surveillance of the relative prevalence of co-circulating strains, and measurements of parameters such as the duration of cross-immunity and their integration with more sophisticated models for the spread of influenza, may allow for more nuanced recommendations for coverage levels that minimize the attack rate of both seasonal and pandemic influenza in the future. Such consideration may prove useful until the development of a universal vaccine that could provide long-duration strain-transcending immunity [67, 68].

## Supporting information

S1 Text Model robustness. We have checked the robustness of our model by determining how the results are affected by introduction of a number of changes: addition of superinfection; addition of age structure; alternative introduction of cross-immunity into the model; addition of seasonality; introduction of the two strains at the same time (at *t* = 0) but at different frequencies; increase in *R*_0_; and addition of the third strain. These changes did not alter the basic result shown in Fig 4—for a large range of parameters the lowest attack rate occurs at intermediate levels of vaccine coverage.
(PDF)

## References

[1] Thompson WW, Shay DK, Weintraub E, Brammer L, Bridges CB, Cox NJ, et al Influenza-associated hospitalizations in the United States. JAMA. 2004;292(11):1333–40. doi: 10.1001/jama.292.11.1333 15367555 10.1001/jama.292.11.1333

[2] US CDC. Vaccination Influenza Vaccination: A Summary for Clinicians. Accessed on January 2017. http://www.cdc.gov/flu/professionals/vaccination/vax-summary.htm

[3] Davenport FM. Inactivated influenza virus vaccines. Past, present, and future. Am Rev Respir Dis. 1961;83(2)Pt 2:146–56. doi: 10.1164/arrd.1961.83.2P2.146 13719955 10.1164/arrd.1961.83.2P2.146

[4] Monto AS, Davenport FM, Napier JA, Francis T Jr. Modification of an outbreak of influenza in Tecumseh, Michigan by vaccination of schoolchildren. J Infect Dis. 1970;122(1):16–25. doi: 10.1093/infdis/122.1-2.16 5433709 10.1093/infdis/122.1-2.16

[5] Loeb M, Russell ML, Moss L, Fonseca K, Fox J, Earn DJD, et al Effect of influenza vaccination of children on infection rates in Hutterite communities: a randomized trial. JAMA. 2010;303(10):943–50. doi: 10.1001/jama.2010.250 20215608 10.1001/jama.2010.250

[6] Reichert TA, Sugaya N, Fedson DS, Glezen WP, Simonsen L, Tashiro M. The Japanese experience with vaccinating schoolchildren against influenza. N Engl J Med. 2001;344(12):889–96. doi: 10.1056/NEJM200103223441204 11259722 10.1056/NEJM200103223441204

[7] Osterholm MT, Kelley NS, Sommer A, Belongia EA. Efficacy and effectiveness of influenza vaccines: a systematic review and meta-analysis. Lancet Infect Dis. 2012;12(1):36–44. doi: 10.1016/S1473-3099(11)70295-X 22032844 10.1016/S1473-3099(11)70295-X

[8] Belongia EA, Simpson MD, King JP, Sundaram ME, Kelley NS, Osterholm MT, et al Variable influenza vaccine effectiveness by subtype: a systematic review and meta-analysis of test-negative design studies. Lancet Infect Dis. 2016;16(8):942–51. doi: 10.1016/S1473-3099(16)00129-8 27061888 10.1016/S1473-3099(16)00129-8

[9] 2017-2018 Influenza Season Week 2 ending January 13, 2018. Accessed on April 2018. https://www.cdc.gov/flu/weekly/weeklyarchives2017-2018/Week02.htm;.

[10] US CDC for 2014-2015 Influenza Season. Accessed on January 2017. For the weeks of 2014: https://www.cdc.goeekly/weeklyarchives2014-2015/week44.htm,…, https://www.cdc.gov/flu/weekly/weeklyarchives2014-2015/week53.htm. For the weeks of 2015: https://www.cdc.gov/flu/weekly/weeklyarchives2014-2015/week1.htm,…, https://www.cdc.gov/flu/weekly/weeklyarchives2014-2015/week20.htm

[11] Flannery B, Zimmerman RK, Gubareva LV, Garten RJ, Chung JR, Nowalk MP, et al Enhanced Genetic Characterization of Influenza A(H3N2) Viruses and Vaccine Effectiveness by Genetic Group, 2014-2015. J Infect Dis. 2016;214(7):1010–9. doi: 10.1093/infdis/jiw181 27190176 10.1093/infdis/jiw181 PMC5812259

[12] US CDC. 2007-2008 Influenza Season Week 19, ending May 10, 2008. Accessed on January 2017. http://www.cdc.gov/flu/weekly/weeklyarchives2007-2008/weekly19.htm

[13] Couch RB, Atmar RL, Franco LM, Quarles JM, Wells J, Arden N, et al Antibody correlates and predictors of immunity to naturally occurring influenza in humans and the importance of antibody to the neuraminidase. J Infect Dis. 2013;207(6):974–81. doi: 10.1093/infdis/jis935 23307936 10.1093/infdis/jis935 PMC3633450

[14] Hamilton JR, Sachs D, Lim JK, Langlois RA, Palese P, Heaton NS. Club cells surviving influenza A virus infection induce temporary nonspecific antiviral immunity. Proc Natl Acad Sci U S A. 2016;113(14):3861–6. doi: 10.1073/pnas.1522376113 27001854 10.1073/pnas.1522376113 PMC4833272

[15] Liang S, Mozdzanowska K, Palladino G, Gerhard W. Heterosubtypic immunity to influenza type A virus in mice. Effector mechanisms and their longevity. J Immunol. 1994;152(4):1653–61. 8120375

[16] Kreijtz JHCM, Bodewes R, van Amerongen G, Kuiken T, Fouchier RAM, Osterhaus ADME, et al Primary influenza A virus infection induces cross-protective immunity against a lethal infection with a heterosubtypic virus strain in mice. Vaccine. 2007;25(4):612–20. doi: 10.1016/j.vaccine.2006.08.036 17005299 10.1016/j.vaccine.2006.08.036

[17] Kreijtz JHCM, Bodewes R, van den Brand JMA, de Mutsert G, Baas C, van Amerongen G, et al Infection of mice with a human influenza A/H3N2 virus induces protective immunity against lethal infection with influenza A/H5N1 virus. Vaccine. 2009;27(36):4983–9. doi: 10.1016/j.vaccine.2009.05.079 19538996 10.1016/j.vaccine.2009.05.079

[18] Hillaire MLB, van Trierum SE, Kreijtz JHCM, Bodewes R, Geelhoed-Mieras MM, Nieuwkoop NJ, et al Cross-protective immunity against influenza pH1N1 2009 viruses induced by seasonal influenza A (H3N2) virus is mediated by virus-specific T-cells. J Gen Virol. 2011;92(Pt 10):2339–49. doi: 10.1099/vir.0.033076-0 21653752 10.1099/vir.0.033076-0

[19] Zarnitsyna VI, Handel A, McMaster SR, Hayward SL, Kohlmeier JE, Antia R. Mathematical Model Reveals the Role of Memory CD8 T Cell Populations in Recall Responses to Influenza. Front Immunol. 2016;7:165 doi: 10.3389/fimmu.2016.00165 27242779 10.3389/fimmu.2016.00165 PMC4861172

[20] Ferguson NM, Galvani AP, Bush RM. Ecological and immunological determinants of influenza evolution. Nature. 2003;422(6930):428–33. doi: 10.1038/nature01509 12660783 10.1038/nature01509

[21] Tria F, Lässig M, Peliti L, Franz S. A minimal stochastic model for influenza evolution. Journal of Statistical Mechanics: Theory and Experiment. 2005; p. P07008.

[22] Koelle K, Khatri P, Kamradt M, Kepler TB. A two-tiered model for simulating the ecological and evolutionary dynamics of rapidly evolving viruses, with an application to influenza. J R Soc Interface. 2010;7(50):1257–74. doi: 10.1098/rsif.2010.0007 20335193 10.1098/rsif.2010.0007 PMC2894885

[23] Sonoguchi T, Naito H, Hara M, Takeuchi Y, Fukumi H. Cross-subtype protection in humans during sequential, overlapping, and/or concurrent epidemics caused by H3N2 and H1N1 influenza viruses. J Infect Dis. 1985;151(1):81–8. doi: 10.1093/infdis/151.1.81 3965596 10.1093/infdis/151.1.81

[24] Bedford T, Rambaut A, Pascual M. Canalization of the evolutionary trajectory of the human influenza virus. BMC Biol. 2012;10:38 doi: 10.1186/1741-7007-10-38 22546494 10.1186/1741-7007-10-38 PMC3373370

[25] Koelle K, Cobey S, Grenfell B, Pascual M. Epochal evolution shapes the phylodynamics of interpandemic influenza A (H3N2) in humans. Science. 2006;314(5807):1898–903. doi: 10.1126/science.1132745 17185596 10.1126/science.1132745

[26] Recker M, Pybus OG, Nee S, Gupta S. The generation of influenza outbreaks by a network of host immune responses against a limited set of antigenic types. Proc Natl Acad Sci U S A. 2007;104(18):7711–6. doi: 10.1073/pnas.0702154104 17460037 10.1073/pnas.0702154104 PMC1855915

[27] Gupta S, Maiden MCJ, Feavers IM, Nee S, May RM, Anderson RM. The maintenance of strain structure in populations of recombining infectious agents. Nature Medicine. 1996;2:437–442. doi: 10.1038/nm0496-437 8597954 10.1038/nm0496-437

[28] Lipsitch M. Vaccination against colonizing bacteria with multiple serotypes. Proc Natl Acad Sci U S A. 1997;94(12):6571–6. doi: 10.1073/pnas.94.12.6571 9177259 10.1073/pnas.94.12.6571 PMC21091

[29] Wearing HJ, Rohani P. Ecological and immunological determinants of dengue epidemics. Proc Natl Acad Sci U S A. 2006;103(31):11802–7. doi: 10.1073/pnas.0602960103 16868086 10.1073/pnas.0602960103 PMC1544250

[30] Castillo-Chavez C, Hethcote HW, Andreasen V, Levin SA, Liu WM. Epidemiological models with age structure, proportionate mixing, and cross-immunity. J Math Biol. 1989;27(3):233–58. doi: 10.1007/BF00275810 2746140 10.1007/BF00275810

[31] Andreasen V, Lin J, Levin SA. The dynamics of cocirculating influenza strains conferring partial cross-immunity. J Math Biol. 1997;35(7):825–42. doi: 10.1007/s002850050079 9269738 10.1007/s002850050079

[32] Kermack WO, McKendrick AG. A contribution to the mathematical theory of epidemics. Proc Roy Soc Lond. 1927;115:700–721. doi: 10.1098/rspa.1927.0118

[33] Anderson RM, May RM. Infectious Diseases of Humans Dynamics and Control. Oxford University Press; 1992.

[34] Keeling MJ, Rohani P. Modeling Infectious Diseases in Humans and Animals. Princeton University Press; 2007.

[35] Gog JR, Grenfell BT. Dynamics and selection of many-strain pathogens. Proc Natl Acad Sci U S A. 2002;99(26):17209–14. doi: 10.1073/pnas.252512799 12481034 10.1073/pnas.252512799 PMC139294

[36] Boni MF, Gog JR, Andreasen V, Feldman MW. Epidemic dynamics and antigenic evolution in a single season of influenza A. Proc Biol Sci. 2006;273(1592):1307–16. doi: 10.1098/rspb.2006.3466 16777717 10.1098/rspb.2006.3466 PMC1560306

[37] Kryazhimskiy S, Dieckmann U, Levin SA, Dushoff J. On state-space reduction in multi-strain pathogen models, with an application to antigenic drift in influenza A. PLoS Comput Biol. 2007;3(8):e159 doi: 10.1371/journal.pcbi.0030159 17708677 10.1371/journal.pcbi.0030159 PMC1949840

[38] Mills CE, Robins JM, Lipsitch M. Transmissibility of 1918 pandemic influenza. Nature. 2004;432(7019):904–6. doi: 10.1038/nature03063 15602562 10.1038/nature03063 PMC7095078

[39] Cauchemez S, Valleron AJ, Boëlle PY, Flahault A, Ferguson NM. Estimating the impact of school closure on influenza transmission from Sentinel data. Nature. 2008;452(7188):750–4. doi: 10.1038/nature06732 18401408 10.1038/nature06732

[40] Carrat F, Vergu E, Ferguson NM, Lemaitre M, Cauchemez S, Leach S, et al Time lines of infection and disease in human influenza: a review of volunteer challenge studies. Am J Epidemiol. 2008;167(7):775–85. doi: 10.1093/aje/kwm375 18230677 10.1093/aje/kwm375

[41] Fung ICH, Antia R, Handel A. How to minimize the attack rate during multiple influenza outbreaks in a heterogeneous population. PLoS One. 2012;7(6):e36573 doi: 10.1371/journal.pone.0036573 22701558 10.1371/journal.pone.0036573 PMC3372524

[42] Meeyai A, Praditsitthikorn N, Kotirum S, Kulpeng W, Putthasri W, Cooper BS, et al Seasonal influenza vaccination for children in Thailand: a cost-effectiveness analysis. PLoS Med. 2015;12(5):e1001829; discussion e1001829. doi: 10.1371/journal.pmed.1001829 26011712 10.1371/journal.pmed.1001829 PMC4444096

[43] May RM. Vaccination programmes and herd immunity. Nature. 1982;300(5892):481–3. doi: 10.1038/300481a0 7144901 10.1038/300481a0

[44] McLean AR, Anderson RM. Measles in developing countries. Part II. The predicted impact of mass vaccination. Epidemiol Infect. 1988;100(3):419–42. doi: 10.1017/S0950268800067170 3378585 10.1017/s0950268800067170 PMC2249353

[45] Handel A, Longini IM Jr, Antia R. What is the best control strategy for multiple infectious disease outbreaks? Proc Biol Sci. 2007;274(1611):833–7. doi: 10.1098/rspb.2006.0015 17251095 10.1098/rspb.2006.0015 PMC2093965

[46] Lipsitch M, Cohen T, Murray M, Levin BR. Antiviral Resistance and the Control of Pandemic Influenza. PLoS Med. 2007;4(1):e15 doi: 10.1371/journal.pmed.0040015 17253900 10.1371/journal.pmed.0040015 PMC1779817

[47] Moghadas SM, Bowman CS, Röst G, Wu J. Population-wide emergence of antiviral resistance during pandemic influenza. PLoS ONE. 2008;3(3):e1839 doi: 10.1371/journal.pone.0001839 18350174 10.1371/journal.pone.0001839 PMC2266801

[48] Handel A, Longini IM Jr, Antia R. Antiviral resistance and the control of pandemic influenza: the roles of stochasticity, evolution and model details. J Theor Biol. 2009;256(1):117–25. doi: 10.1016/j.jtbi.2008.09.021 18952105 10.1016/j.jtbi.2008.09.021 PMC2624577

[49] Worby CJ, Wallinga J, Lipsitch M, Goldstein E. Population effect of influenza vaccination under co-circulation of non-vaccine variants and the case for a bivalent A/H3N2 vaccine component. Epidemics. 2017 doi: 10.1016/j.epidem.2017.02.008 28262588 10.1016/j.epidem.2017.02.008 PMC5533618

[50] Antia R, Koella JC. A model of non-specific immunity. J Theor Biol. 1994;168(2):141–50. doi: 10.1006/jtbi.1994.1094 8022194 10.1006/jtbi.1994.1094

[51] Wu T, Hu Y, Lee YT, Bouchard KR, Benechet A, Khanna K, et al Lung-resident memory CD8 T cells (TRM) are indispensable for optimal cross-protection against pulmonary virus infection. J Leukoc Biol. 2014;95(2):215–24. doi: 10.1189/jlb.0313180 24006506 10.1189/jlb.0313180 PMC3896663

[52] McMaster SR, Wilson JJ, Wang H, Kohlmeier JE. Airway-Resident Memory CD8 T Cells Provide Antigen-Specific Protection against Respiratory Virus Challenge through Rapid IFN-*γ* Production. J Immunol. 2015;195(1):203–9. doi: 10.4049/jimmunol.1402975 26026054 10.4049/jimmunol.1402975 PMC4475417

[53] Slütter B, Van Braeckel-Budimir N, Abboud G, Varga SM, Salek-Ardakani S, Harty JT. Dynamics of influenza-induced lung-resident memory T cells underlie waning heterosubtypic immunity. Sci Immunol. 2017;2(7). doi: 10.1126/sciimmunol.aag2031 28783666 10.1126/sciimmunol.aag2031 PMC5590757

[54] Halloran ME, Ferguson NM, Eubank S, Longini IM Jr, Cummings DAT, Lewis B, et al Modeling targeted layered containment of an influenza pandemic in the United States. Proc Natl Acad Sci U S A. 2008;105(12):4639–44. doi: 10.1073/pnas.0706849105 18332436 10.1073/pnas.0706849105 PMC2290797

[55] Fraser C, Donnelly CA, Cauchemez S, Hanage WP, Van Kerkhove MD, Hollingsworth TD, et al Pandemic potential of a strain of influenza A (H1N1): early findings. Science. 2009;324(5934):1557–61. doi: 10.1126/science.1176062 19433588 10.1126/science.1176062 PMC3735127

[56] Park AW, Daly JM, Lewis NS, Smith DJ, Wood JLN, Grenfell BT. Quantifying the impact of immune escape on transmission dynamics of influenza. Science. 2009;326(5953):726–8. doi: 10.1126/science.1175980 19900931 10.1126/science.1175980 PMC3800096

[57] Fonville JM, Wilks SH, James SL, Fox A, Ventresca M, Aban M, et al Antibody landscapes after influenza virus infection or vaccination. Science. 2014;346(6212):996–1000. doi: 10.1126/science.1256427 25414313 10.1126/science.1256427 PMC4246172

[58] Handel A, Longini IM Jr, Antia R. Intervention strategies for an influenza pandemic taking into account secondary bacterial infections. Epidemics. 2009;1(3):185–95. doi: 10.1016/j.epidem.2009.09.001 20161493 10.1016/j.epidem.2009.09.001 PMC2796779

[59] McCullers JA, Huber VC. Correlates of vaccine protection from influenza and its complications. Hum Vaccin Immunother. 2012;8(1):34–44. doi: 10.4161/hv.8.1.18214 22252001 10.4161/hv.8.1.18214

[60] Mina MJ, Klugman KP. The role of influenza in the severity and transmission of respiratory bacterial disease. Lancet Respir Med. 2014;2(9):750–63. doi: 10.1016/S2213-2600(14)70131-6 25131494 10.1016/S2213-2600(14)70131-6 PMC4823014

[61] Smith AM, Huber VC. The Unexpected Impact of Vaccines on Secondary Bacterial Infections Following Influenza. Viral Immunol. 2018;31(2):159–173. doi: 10.1089/vim.2017.0138 29148920 10.1089/vim.2017.0138 PMC5863092

[62] Subramanian R, Graham AL, Grenfell BT, Arinaminpathy N. Universal or Specific? A Modeling-Based Comparison of Broad-Spectrum Influenza Vaccines against Conventional, Strain-Matched Vaccines. PLoS Comput Biol. 2016;12(12):e1005204 doi: 10.1371/journal.pcbi.1005204 27977667 10.1371/journal.pcbi.1005204 PMC5157952

[63] Bui HH, Peters B, Assarsson E, Mbawuike I, Sette A. Ab and T cell epitopes of influenza A virus, knowledge and opportunities. Proc Natl Acad Sci U S A. 2007;104(1):246–51. doi: 10.1073/pnas.0609330104 17200302 10.1073/pnas.0609330104 PMC1765443

[64] van de Sandt CE, Kreijtz JHCM, de Mutsert G, Geelhoed-Mieras MM, Hillaire MLB, Vogelzang-van Trierum SE, et al Human cytotoxic T lymphocytes directed to seasonal influenza A viruses cross-react with the newly emerging H7N9 virus. J Virol. 2014;88(3):1684–93. doi: 10.1128/JVI.02843-13 24257602 10.1128/JVI.02843-13 PMC3911609

[65] Miller MA, Viboud C, Balinska M, Simonsen L. The signature features of influenza pandemics–implications for policy. N Engl J Med. 2009;360(25):2595–8. doi: 10.1056/NEJMp0903906 19423872 10.1056/NEJMp0903906

[66] Skowronski DM, De Serres G, Crowcroft NS, Janjua NZ, Boulianne N, Hottes TS, et al Association between the 2008-09 seasonal influenza vaccine and pandemic H1N1 illness during Spring-Summer 2009: four observational studies from Canada. PLoS Med. 2010;7(4):e1000258 doi: 10.1371/journal.pmed.1000258 20386731 10.1371/journal.pmed.1000258 PMC2850386

[67] Krammer F, Pica N, Hai R, Margine I, Palese P. Chimeric hemagglutinin influenza virus vaccine constructs elicit broadly protective stalk-specific antibodies. J Virol. 2013;87(12):6542–50. doi: 10.1128/JVI.00641-13 23576508 10.1128/JVI.00641-13 PMC3676110

[68] Yassine HM, Boyington JC, McTamney PM, Wei CJ, Kanekiyo M, Kong WP, et al Hemagglutinin-stem nanoparticles generate heterosubtypic influenza protection. Nat Med. 2015;21(9):1065–70. doi: 10.1038/nm.3927 26301691 10.1038/nm.3927

