# Supplementary material for: Intermediate levels of vaccination coverage may minimize seasonal influenza outbreaks
Source: PLoS One. 2018 Jun 26;13(6):e0199674. doi: 10.1371/journal.pone.0199674 (PMC6019388; doi:10.1371/journal.pone.0199674)
Supplement: S1 Text — We have checked the robustness of our model by determining how the results are affected by introduction of a number of changes: addition of superinfection; addition of age structure; alternative introduction of cross-immunity into the model; addition of seasonality; introduction of the two strains at the same time (at t = 0) but at different frequencies; increase in R0; and addition of the third strain. These changes did not alter the basic result shown in Fig 4—for a large range of parameters the lowest attack rate occurs at intermediate levels of vaccine coverage. (PDF) [file pone.0199674.s001.pdf]

Supporting Information

Fig A. Addition of superinfection to the model. We considered the robustness of the model shown in Fig 2 to the addition of superinfection. Individuals infected with either the first (IS) or second (SI) strains can be superinfected with the other strain to give rise to the II state. The rate parameters for the transitions are shown with the corresponding arrows.

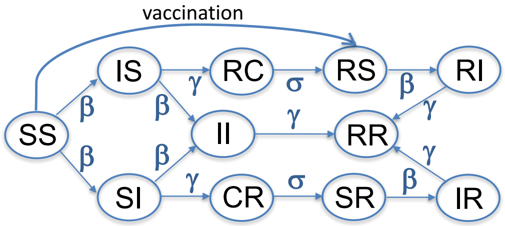

The model equations are:

$$\frac{dSS}{dt} = -\beta(IS + IR + SI + RI + 2II)SS \tag{1}$$

$$\frac{dIS}{dt} = \beta(IS + IR + II)SS - \gamma IS - \beta(SI + RI + II)IS \tag{2}$$

$$\frac{dSI}{dt} = \beta(SI + RI + II)SS - \gamma SI - \beta(IS + IR + II)SI \tag{3}$$

$$\frac{dRC}{dt} = \gamma IS - \sigma RC \tag{4}$$

$$\frac{dCR}{dt} = \gamma SI - \sigma CR \tag{5}$$

$$\frac{dRS}{dt} = \sigma RC - \beta(SI + RI + II)RS \tag{6}$$

$$\frac{dSR}{dt} = \sigma CR - \beta(IS + IR + II)SR \tag{7}$$

$$\frac{dRI}{dt} = \beta(SI + RI + II)RS - \gamma RI \tag{8}$$

$$\frac{dIR}{dt} = \beta(IS + IR + II)SR - \gamma IR \tag{9}$$

$$\frac{dRR}{dt} = \gamma(IR + RI + II) \tag{10}$$

$$\frac{dII}{dt} = \beta(SI + RI + II)IS + \beta(IS + IR + II)SI - \gamma II \tag{11}$$

Fig B. Addition of age structure to the model. We considered the robustness of the model shown in Fig 2 to the addition of simple age structure along the lines previously described [1]. Two age populations, children (subscript "C", top scheme) and adults (subscript "A", bottom scheme) are considered and have different parameters. We have four transmission terms: β_{AA} defined as the rate of transmission from adults to adults; β_{AC} from adults to children; β_{CC} from children to children; and β_{CA} from children to adults. Recovery rates for infections of children and adults are γ_C and γ_A, respectively.

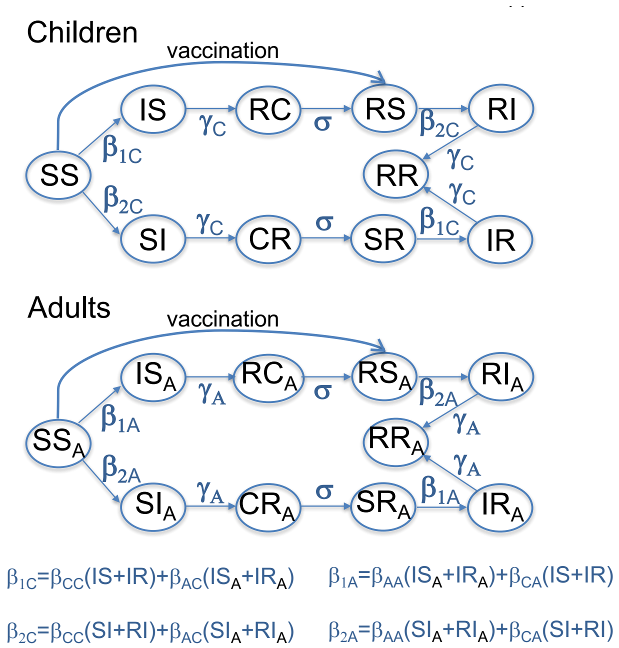

The model equations are:

$$\frac{dSS}{dt} = -(\beta_{1CC}(IS+IR)+\beta_{2CC}(SI+RI)+\beta_{1AC}(IS_A+IR_A)+\beta_{2AC}(SI_A+RI_A))SS \quad (12)$$

$$\frac{dIS}{dt} = (\beta_{1CC}(IS+IR)+\beta_{1AC}(IS_A+IR_A))SS-\gamma IS \quad (13)$$

$$\frac{dSI}{dt} = (\beta_{2CC}(SI+RI)+\beta_{2AC}(SI_A+RI_A))SS-\gamma SI \quad (14)$$

$$\frac{dRC}{dt} = \gamma IS-\sigma RC \quad (15)$$

$$\frac{dCR}{dt} = \gamma SI-\sigma CR \quad (16)$$

$$\frac{dRS}{dt} = \sigma RC-(\beta_{2CC}(SI+RI)+\beta_{2AC}(SI_A+RI_A))RS \quad (17)$$

$$\frac{dSR}{dt} = \sigma CR-(\beta_{1CC}(IS+IR)+\beta_{1AC}(IS_A+IR_A))SR \quad (18)$$

$$\frac{dRI}{dt} = (\beta_{2CC}(SI+RI)+\beta_{2AC}(SI_A+RI_A))RS-\gamma RI \quad (19)$$

$$\frac{dIR}{dt} = (\beta_{1CC}(IS+IR)+\beta_{1AC}(IS_A+IR_A))SR-\gamma IR \quad (20)$$

$$\frac{dRR}{dt} = \gamma(IR+RI) \quad (21)$$

$$\frac{dSS_A}{dt} = -(\beta_{1AA}(IS_A+IR_A)+\beta_{2AA}(SI_A+RI_A)+\beta_{1CA}(IS+IR)+\beta_{2CA}(SI+RI))SS_A \quad (22)$$

$$\frac{dIS_A}{dt} = (\beta_{1AA}(IS_A+IR_A)+\beta_{1CA}(IS+IR))SS_A-\gamma_A IS_A \quad (23)$$

$$\frac{dSI_A}{dt} = (\beta_{2AA}(SI_A+RI_A)+\beta_{2CA}(SI+RI))SS_A-\gamma_A SI_A \quad (24)$$

$$\frac{dRC_A}{dt} = \gamma_A IS_A-\sigma RC_A \quad (25)$$

$$\frac{dCR_A}{dt} = \gamma_A SI_A-\sigma CR_A \quad (26)$$

$$\frac{dRS_A}{dt} = \sigma RC_A-(\beta_{2AA}(SI_A+RI_A)+\beta_{2CA}(SI+RI))RS_A \quad (27)$$

$$\frac{dSR_A}{dt} = \sigma CR_A-(\beta_{1AA}(IS_A+IR_A)+\beta_{1CA}(IS+IR))SR_A \quad (28)$$

$$\frac{dRI_A}{dt} = (\beta_{2AA}(SI_A+RI_A)+\beta_{2CA}(SI+RI))RS_A-\gamma_A RI_A \quad (29)$$

$$\frac{dIR_A}{dt} = (\beta_{1AA}(IS_A+IR_A)+\beta_{1CA}(IS+IR))SR_A-\gamma_A IR_A \quad (30)$$

$$\frac{dRR_A}{dt} = \gamma_A(IR_A+RI_A) \quad (31)$$

Fig C. Different introduction of cross-immunity into the model. We considered the robustness of the model shown in Fig 2 to changes in the term for cross-immunity along the lines previously described [2]. We let a fraction σ of individuals infected with one strain to have long-term cross-immunity to the second strain and the remaining fraction $(1-\sigma)$ to have no cross-immunity.

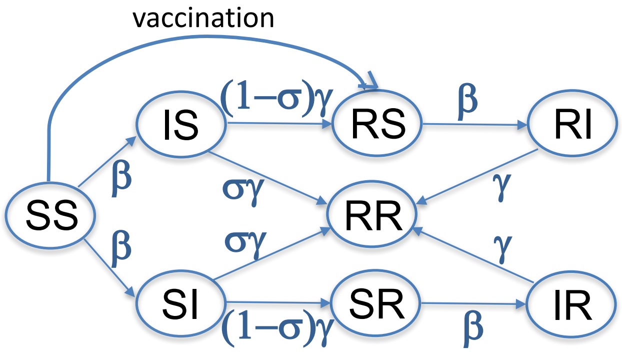

The model equations are:

$$\frac{dSS}{dt} = -\beta(IS + IR + SI + RI)SS \tag{32}$$

$$\frac{dIS}{dt} = \beta(IS + IR)SS - \gamma IS \tag{33}$$

$$\frac{dSI}{dt} = \beta(SI + RI)SS - \gamma SI \tag{34}$$

$$\frac{dRS}{dt} = (1-\sigma)\gamma IS - \beta(SI + RI)RS \tag{35}$$

$$\frac{dSR}{dt} = (1-\sigma)\gamma SI - \beta(IS + IR)SR \tag{36}$$

$$\frac{dIR}{dt} = \beta(IS + IR)SR - \gamma IR \tag{37}$$

$$\frac{dRI}{dt} = \beta(SI + RI)RS - \gamma RI \tag{38}$$

$$\frac{dRR}{dt} = \gamma(IR + \sigma IS + RI + \sigma SI)) \tag{39}$$

Fig D. Robustness to the addition of superinfection. We allowed for superinfection as described in Fig A. We plotted how the total attack rate depends on the vaccine coverage for different durations of cross-immunity (Panel A) and the degrees of dominance of the first strain (Panel B). The addition of superinfection did not alter the basic result shown in Fig 4 – for a large range of parameters the lowest attack rate occurs at intermediate levels of vaccine coverage. Parameters as in Fig 4.

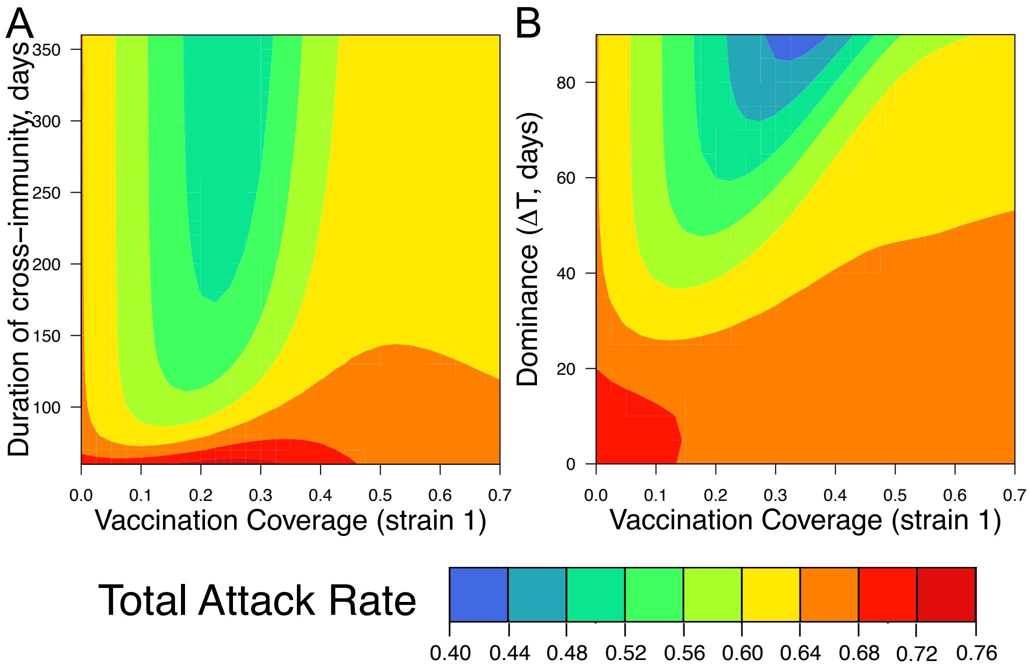

Fig E. Robustness to the addition of age structure. We added age structure as described in Figure B. We plotted how the total attack rate depends on the vaccine coverage for different durations of cross-immunity (Panel A) and the degrees of dominance of the first strain (Panel B). The addition of age-structure did not alter the basic result shown in Fig 4 – for a large range of parameters the lowest attack rate occurs at intermediate levels of vaccine coverage. Parameters for age structure as in [1]: $\beta_{AA} = 0.33; \beta_{AC} = 0.033; \beta_{CC} = 0.79; \beta_{CA} = 0.079$, and recovery rates $\gamma_A = 1/4.8$ day^{-1} and $\gamma_C = 1/8$ day^{-1}. Fraction of adults was set to 0.76 (population with 24% of children). Other parameters as in Fig 4.

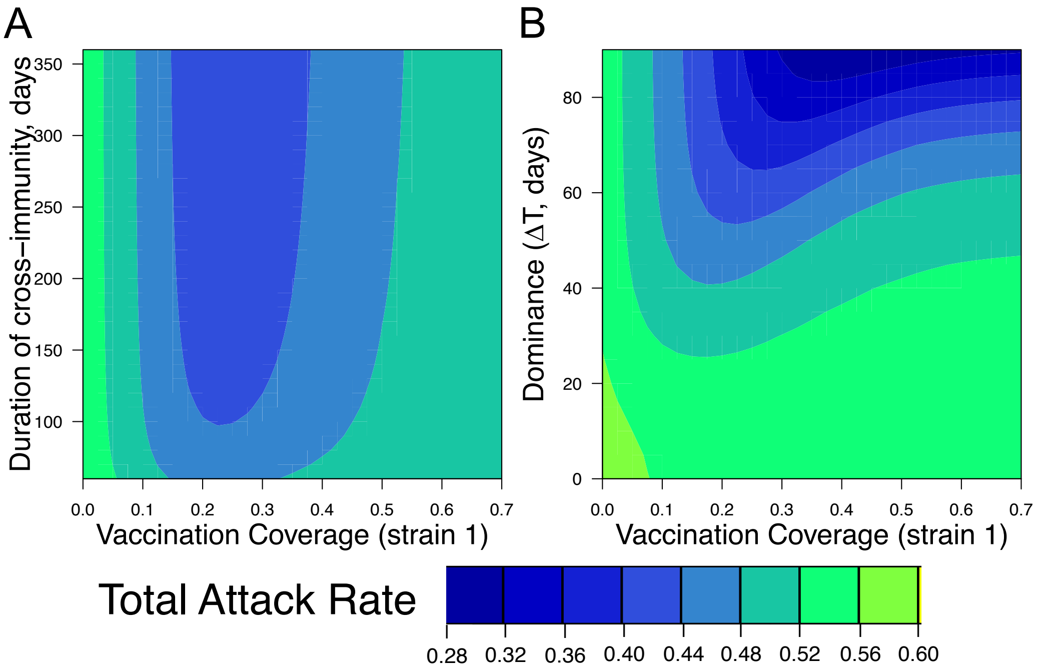

Fig F. Robustness to the changes in the terms for cross-immunity. We introduced cross-immunity as described in Fig C. We plotted how the total attack rate depends on the vaccine coverage for different levels of cross-immunity (Panel A) and the degrees of dominance of the first strain (Panel B). The changes in terms for cross-immunity did not alter the basic result shown in Fig 4 – for a large range of parameters the lowest attack rate occurs at intermediate levels of vaccine coverage. Parameters: σ=0.95, other parameters as in Fig 4.

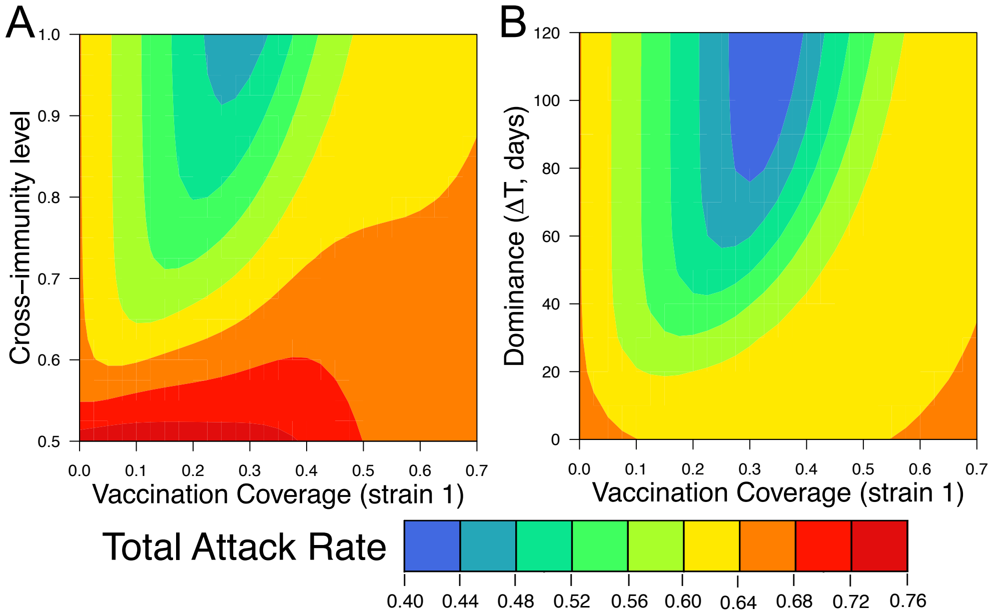

Fig G. Robustness to the addition of seasonality. Seasonality was added to the model by changing the parameter β as a function of time, so that $\beta(t) = \beta(1 + \beta_s \sin(2\pi t/365))$, where $\beta_s = 0.25$. We plotted how the total attack rate depends on the vaccine coverage for different durations of cross-immunity (Panel A) and the degrees of dominance of the first strain (Panel B). The addition of seasonality did not alter the basic result shown in Fig 4 – for a large range of parameters the lowest attack rate occurs at intermediate levels of vaccine coverage. Other parameters as in Fig 4.

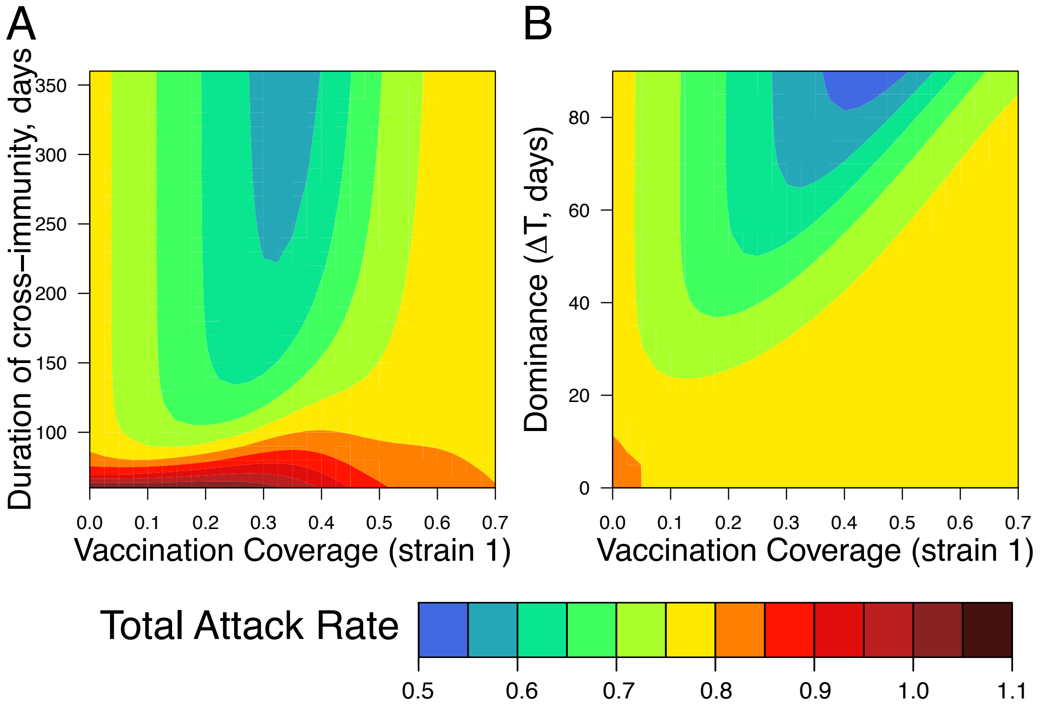

Fig H. Robustness to changes in the introduction of the two strains. Previously we introduced the subdominant strain at a later time, and here we considered the effect of introducing the two strains at the same time (at $t = 0$) but at different frequencies. The degree of dominance is now defined by the relative frequency of the two strains. We plotted how the total attack rate depends on the vaccine coverage for different durations of cross-immunity (Panel A) and the degrees of dominance (ratio of strain prevalences at $t = 0$) of the first strain (Panel B). The change to the introduction of the strains did not alter the basic result shown in Fig 4 – for a large range of parameters the lowest attack rate occurs at intermediate levels of vaccine coverage. The total prevalence of both strain at $t = 0$ was set to $= 4 \times 10^{-4}$, and the ratio of the initial two strains prevalences was altered. For Panel A the initial prevalence of the second strain is 2.5%. Other parameters as in Fig 4.

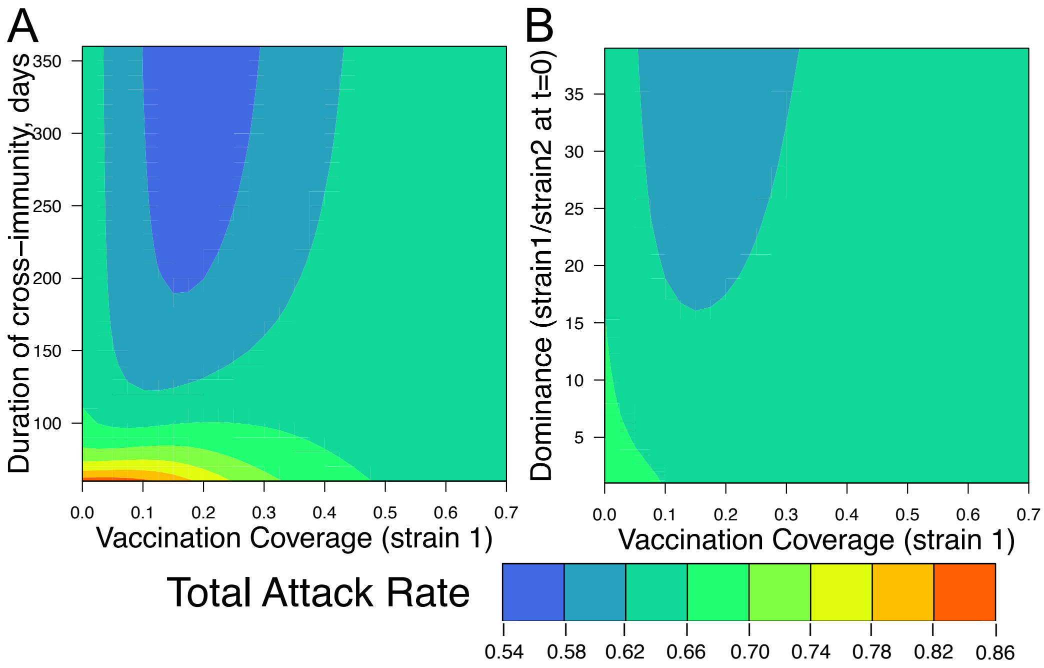

Fig I in S1 Text. Robustness to increase in R_0. We plotted how the total attack rate depends on the vaccine coverage for different durations of cross-immunity (Panel A) and the degrees of dominance of the first strain (Panel B) when R_0 was increased to 2. The increase in R_0 did not alter the basic result shown in Fig 4 – for a large range of parameters the lowest attack rate occurs at intermediate levels of vaccine coverage. Other parameters as in Fig 4.

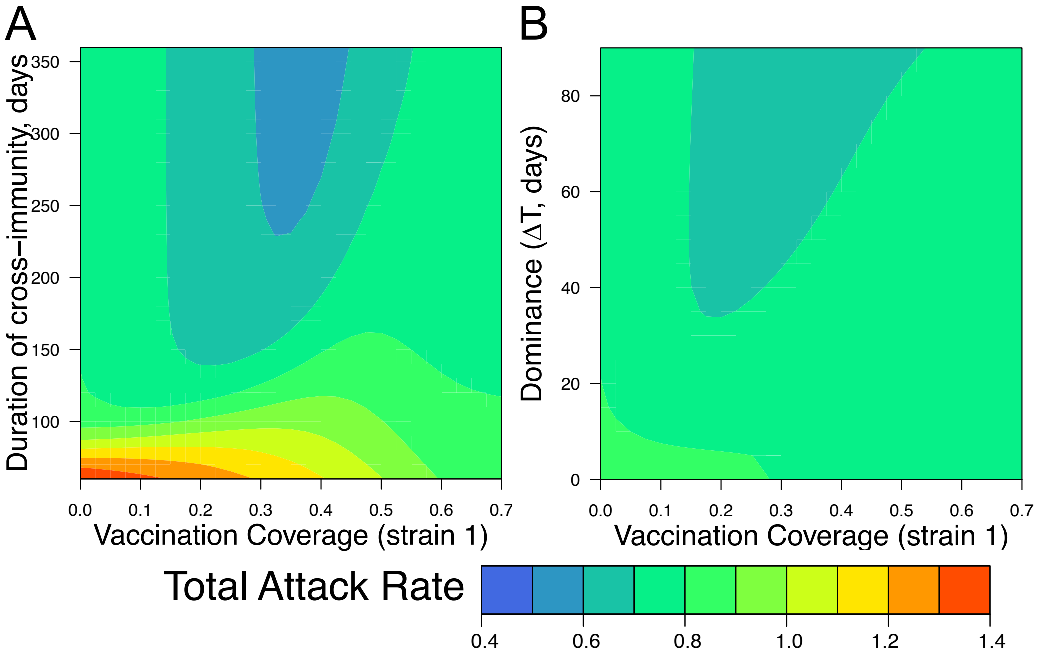

Fig J in S1 Text. Scheme for SIR-based model with three strains. Twenty six different states corresponding to model variables characterize the status of individuals with respect to the first, second and third strains of the virus. The first, second and third letters in the three-letter code show the individual's status of infection with respect to the first and second strain. We use the conventional notation "S" for susceptible, "I" for infected, and "R" for recovered with long-term immunity and "C" for recovered with short-term cross-immunity.

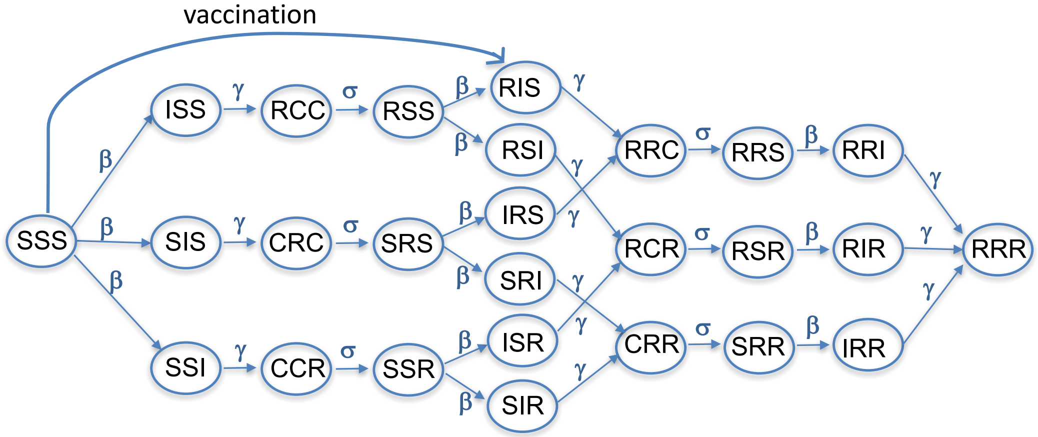

Fig K in S1 Text. Robustness to the addition of the third strain. We considered a case with three co-circulated strains with one strain included in the vaccine and two strains not targeted by a vaccine. We plotted how the total attack rate depends on the vaccine coverage for different durations of cross-immunity (Panel A) and the degrees of dominance of the first strain (Panel B). The addition of the third strain did not alter the basic result shown in Fig 4 – for a large range of parameters the lowest attack rate occurs at intermediate levels of vaccine coverage. Second and third strains were added simultaneously at 60 days (panel A) and at indicated times ΔT (panel B) following introduction of the first strain. The sum of their initial prevalences at the moment of introduction was kept the same as for the second strain in two-strain model (1)-(10). Other parameters as in Fig 4.

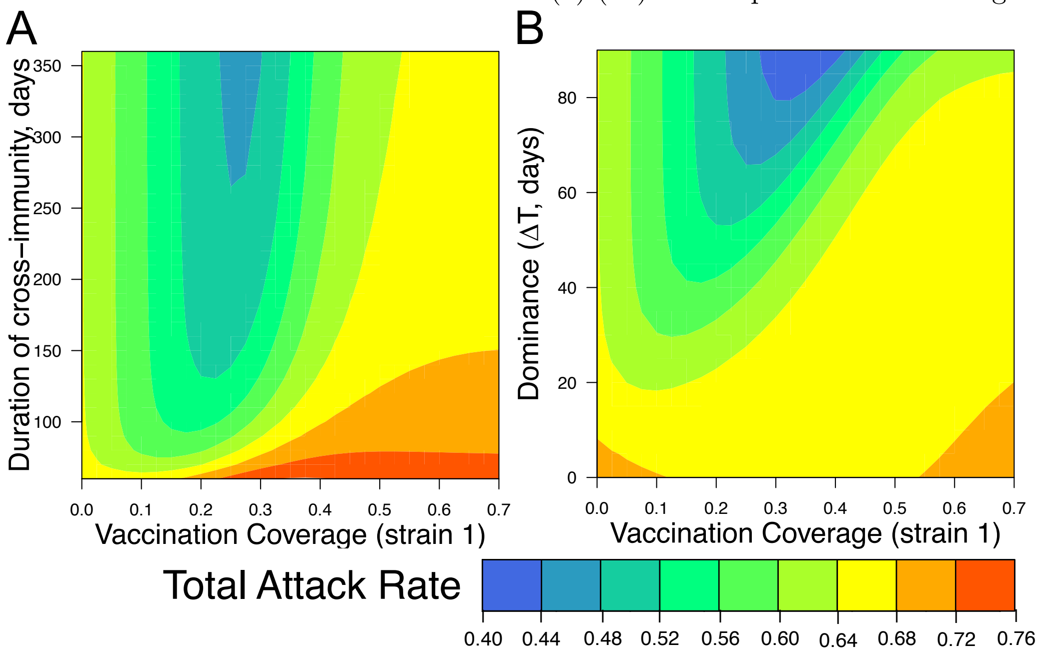

References

[1] I. C.-H. Fung, R. Antia, and A. Handel. How to minimize the attack rate during multiple influenza outbreaks in a heterogeneous population. *PLoS One*, 7(6):e36573, 2012.

[2] K. Koelle, P. Khatri, M. Kamradt, and T. B. Kepler. A two-tiered model for simulating the ecological and evolutionary dynamics of rapidly evolving viruses, with an application to influenza. *J R Soc Interface*, 7(50):1257–74, Sep 2010.
